# Performing corneal crosslinking under local anaesthesia in patients with Down syndrome

**DOI:** 10.1007/s10792-017-0535-1

**Published:** 2017-04-19

**Authors:** Nienke Soeters, Esmée Bennen, Robert P. L. Wisse

**Affiliations:** Utrecht Cornea Research Group, Department of Ophthalmology, University Medical Center Utrecht, HP E03.136, Heidelberglaan 100, 3508 GX Utrecht, The Netherlands

**Keywords:** Down syndrome, Keratoconus, Corneal crosslinking, CXL, Local anaesthesia

## Abstract

**Purpose:**

To report on the ability to perform corneal crosslinking (CXL) under local anaesthesia for the treatment of keratoconus in patients with Down syndrome.

**Methods:**

Nine eyes of seven patients with both keratoconus and Down syndrome were scheduled for an epithelium-off CXL procedure under local anaesthesia. Exclusion criteria were a corneal thickness under 400 µm and the presence of corneal scars. A standardized clinical decision tool was used to estimate patient cooperation and the likelihood for a successful procedure under local rather than general anaesthesia.

**Results:**

In seven eyes, the CXL was completed successfully. The treatment was aborted in two eyes due to insufficient corneal thickness (<400 µm) prior to ultraviolet-A irradiation, even after employing hypoosmolar riboflavin. No adverse events occurred post-operatively, except for one case of delayed epithelial healing (23 days).

**Conclusions:**

With a proper patient selection, CXL under local anaesthesia can be achieved in patients with Down syndrome.

## Introduction

Keratoconus has long since been linked with Down syndrome [1]. Reports show a 0.5–15% incidence of keratoconus in patients with Down syndrome, which is much higher than in the general population (1:2000) [2–6]. Keratoconus can be detected at earlier stages with corneal topography, a reliable instrument for screening and diagnosis in patients with Down syndrome [7–9].

The role of trisomy 21 in developing keratoconus remains somewhat unclear. A nonparametric linkage analysis suggested that a gene on chromosome 21 could be related to keratoconus, but this finding was never confirmed [10]. Genetic studies have not yet deciphered the complex genetic architecture of keratoconus. This is perhaps in part due to differential distribution of the risk loci among ethnic populations or the relatively low contribution of genetic variants in developing keratoconus [11].

Patients with Down syndrome often do not complain about their vision. Instead, the ailment is typically noticed by others in their environment, resulting in a diagnosis in a more advanced disease state. The management of keratoconus in patients with Down syndrome varies, depending on the severity of keratoconus and the degree of Down syndrome characteristics. Performing a corneal transplantation in patients with Down syndrome entails considerable risks, and this surgery has a worse prognosis than other patients with keratoconus [12, 13]. Therefore, it is desirable to halt keratoconus progression in an earlier stage and greatly minimize the need for corneal surgery. Corneal crosslinking (CXL) is a minimally invasive procedure that has the potential to slow keratoconus progression and prevent the development of keratoconus into stages where patients become dependent on rigid (scleral) contact lenses or corneal grafting procedures for an adequate visual acuity and quality of life [14, 15]. CXL has an attractive safety profile: it is easy to perform under local anaesthesia, it has few side effects, and it has a low rate of vision threatening complications such as keratitis or corneal haze formation [16].

Patients with Down syndrome show higher risks during general anaesthesia (bradycardia, natural airway obstruction, difficult intubation, post-intubation croup, and bronchospasm); therefore, it is preferred to perform CXL under local anaesthesia [17–19].

Here, we report on the potential to perform CXL procedures under local anaesthesia in patients with Down syndrome, our standardized clinical decision tool, and the outcomes of these treatments.

## Patients and methods

The study was a prospective case series of patients with Down syndrome and keratoconus scheduled for epithelium-off CXL under local anaesthesia at the University Medical Centre, Utrecht. The study was approved by the University Medical Center Utrecht Ethics Review Board, who judged that our research (which was the collection of data of an already scheduled procedure, not performed in a trial) inferred no additional risks for the patient and waived the need for a written informed consent. All patients and their parents/legal representatives were properly informed by their medical specialist, and they consented with the CXL procedure. The treatments were performed according to the highest standards of care and in accordance with the Declaration of Helsinki and local laws regarding research using human subjects.

A full ophthalmic evaluation was done, including the assessment of uncorrected (UDVA) and corrected (CDVA) distance visual acuity, manifest refraction, corneal topography (Pentacam HR; Oculus, Wetzlar, Germany), slit-lamp examination, and dilated fundoscopy. All patients were asked to remove their contact lenses for 2 week prior to the measurements. Exclusion criteria for CXL treatment were a corneal thickness <400 µm prior to UV irradiation and the presence of a corneal scar.

The diagnosis of Down syndrome was apparent in all cases. Furthermore, a semi-structured approach was used to assess patient compliance based on items covering three domains. The decision tool was completed by both the optometrist and ophthalmologist and discussed together after the patient underwent a trial-position in the treatment room. The first domain of the decision tool comprised a general assessment: are spectacles tolerated, is eye contact being made, is there verbal communication, are slit-lamp examination and topography possible? The second domain assessed ability of patients to undergo the treatment: are there abrupt movements of the head or body, can anaesthetic eye drops be tolerated, can eyelid touch be tolerated, is there the ability to be placed in a supine position and fixate on a light for 5 min? The third domain estimated post-procedure compliance: can the patient follow instructions adequately, can they refrain from eye rubbing, are parents or institutional caregivers competent and supporting of treatment? See the standardized clinical decision tool in Table 1.

**Table 1:**
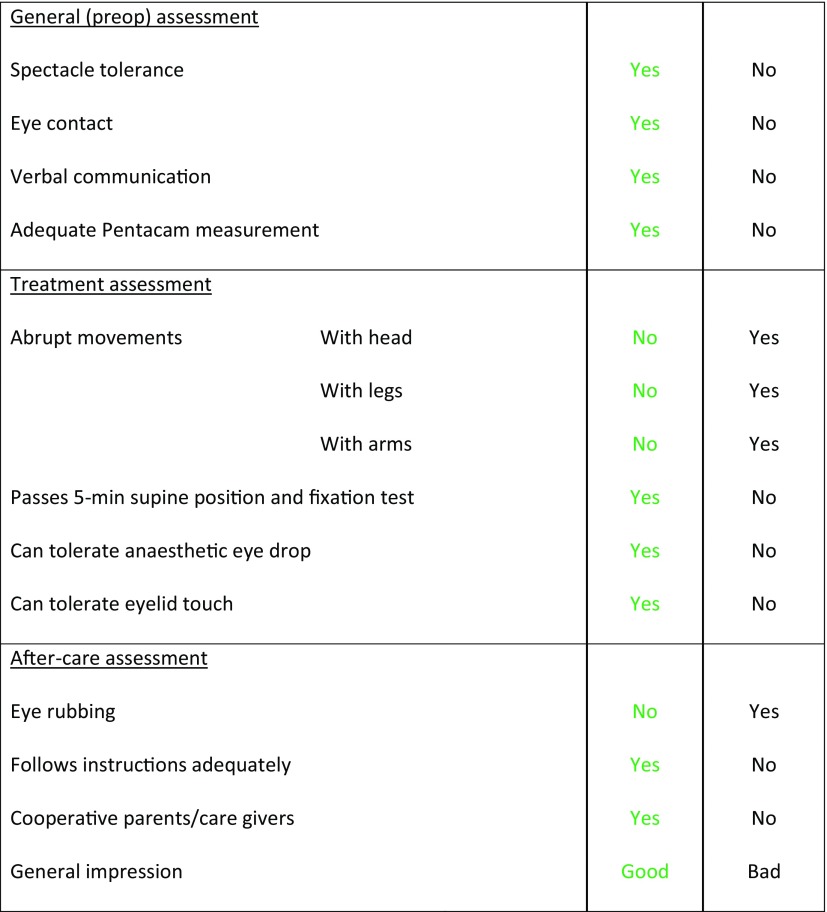
Standardized clinical decision tool to judge the subject suitability of Down syndrome patients for a corneal crosslinking treatment under local anaesthesia

### Procedure

An epithelium-off CXL was performed following the ‘Dresden protocol’ [15, 20]. After local anaesthetics (oxybuprocaine 0.4% and tetracaine 1%) were administered, the epithelium was removed with a blunt spatula at the central 9 mm of the cornea. Isotonic riboflavin drops (0.1%, Innocross-R) with 20% dextran were applied to the cornea every 3 min for 30 min. After this phase, pachymetry measurements were taken. When pachymetry was <400 µm, hypoosmolar riboflavin drops were added every 20 s for 5 min and repeated twice when necessary to reach 400 µm. During the 30 min of ultraviolet-A (UVA) irradiation (UV-X 1000, Peschke Meditrade GmbH, Switzerland), riboflavin was applied on the cornea every 5 min. A bandage contact lens was placed (Purevision, Bausch & Lomb). Antibiotic eye drops (chloramphenicol preservative free 4 mg/ml, BID for 1 month) and pain medication were prescribed (paracetamol 1000 mg QID, diclofenac 50 mg TID). After the epithelium was healed and the bandage contact lens was removed, topical steroids (fluorometholone 1 mg/ml, BID) were started and continued for 3 weeks. Follow-up measurements were taken after 2 days and 1 week to confirm epithelial healing, and then after 1, 3, 6 months, and 1 year. A visit included assessment of UDVA/CDVA, manifest refraction, corneal topography, and slit-lamp examination.

## Results

A total of nine eyes of seven patients with Down syndrome were scheduled for CXL for the treatment of keratoconus between 2011 and 2015. Mean age at the time of treatment was 24 years (range 15–34), 29% were male, and mean follow-up was 26 months (range 12–48). On average, the UDVA was 0.3 Snellen decimal (measured in 3 out of 9 eyes), the CDVA (Snellen decimal) was 0.35 ± 0.18, and refractive astigmatism was −3.00 D ± 2.2. The mean maximal keratometry (*K*_max_) value was 55.6 ± 5.0 D. The mean amount of corneal astigmatism was 3.2 ± 1.7 D, and the mean pre-CXL pachymetry was 447 ± 52 µm. Slit-lamp examinations were feasible in all subjects and revealed no signs of ocular allergies of atopic conjunctivitis. Table 2 gives a detailed overview of the baseline characteristics per patient and post-CXL outcomes. All patients scored ‘positive’ on all items of the standardized clinical decision tool in Table 1, except for patient 6 who had an inadequate Pentacam measurement.

**Table 2:** Patient characteristics (Down syndrome and corneal crosslinking for keratoconus)

Pt	Eye	Age (years)	Sex	Optical correction	Keratoconus stage^a^	CXL completed	Visual acuity (decimal)			Thinnest pachymetry (µm)		Maximal keratometry (D)			
							Preop	6 Mo	12 Mo	Preop	12 Mo	Preop	1 Mo	6 Mo	12 Mo
1	OD	20	F	Scleral lenses	2	No	0.5+ cc	n/a	n/a	384	264	59.6	n/a	n/a	85.5
	OS				2	Yes	0.30 cc	0.40 cc	0.30 cc	390	386	61.1	59.6	60.3	60.5
2	OS	31	F	Spectacles	2	No	0.30= sc	0.30 cc	n/a	419	404	51.3	n/a	51.0	n/a
3	OD	20	M	Spectacles	2	Yes	0.30 ec	0.10 cc	0.03 cc	434	414	63.3	63.1	61.2	61.0
4	OD	15	F	Spectacles	2	Yes	0.50 ec	0.50 cc	0.60 cc	519	505	53.5	n/a	53.1	52.0
	OS				2	Yes	0.50 ec	0.55 cc	0.55 cc	498	484	53.5	52.5	n/a	52.0
5	OS	34	M	None	1	Yes	0.60 sc	0.50 sc	0.65 sc	434	443	51.8	54.3	52.8	56.1
6	OS	21	F	Spectacles	n/a	Yes	n/a	n/a	n/a	n/a	n/a	n/a	n/a	n/a	n/a
7	OD	24	F	Scleral lenses	1	Yes	0.20 cc	0.20 cc	0.20 cc	498	464	50.5	49.9	50.2	50.8

*Pt* patient, *CXL* corneal crosslinking, *Mo* months, *F* female, *M* male, *cc* with refraction, *sc* without correction, *ec* with spectacles, *n/a* not available

^a^Based on Amsler Krumeich classification

All patients were scheduled for CXL under local anaesthesia. Two patients were treated bilaterally, with a 3-month interval. In the other five patients, the fellow eye was unsuitable for CXL due to the presence of a hydrops (patient 3 and 5), the absence of keratoconus (patient 6 and 7), or a cornea of insufficient thickness (patient 2). All patients were examined within a week after treatment. No short-term post-operative complications occurred in any patient, except for a delayed epithelial healing in patient 2 (23 days). In three eyes, the corneal thickness was <400 µm after isotonic riboflavin instillation and additional hypoosmolar riboflavin drops were applied. Two of these eyes remained too thin before the start of UVA radiation and the procedure was aborted. In patient 6, Pentacam measurements were unreliable and neither keratometry readings nor pachymetry were interpretable. During treatment, pachymetry was 414 µm prior to UVA irradiation in this patient. Patient 5 showed a *K*_max_ increase from 51.8 to 56.1 D after 1 year, which decreased to 50.6 D at the 3-year follow-up.

For patient 4, a 16-year-old girl, fixating on the blue light in the UVA-lamp proved to be very tough. To solve this problem, her father held his tablet showing a video behind the UVA-lamp to effectively maintain fixation and prevent abrupt eye movements.

## Discussion

This study reports the feasibility of a crosslinking procedure in patients with Down syndrome under local rather than general anaesthesia. Lack of cooperation was not an issue in any of the cases and the treatment was completed in 7 of 9 eyes. No adverse events were encountered during treatment or in the follow-up period, apart from one case of delayed epithelial healing. A semi-structured assessment to aid in patient selection is therefore proposed.

A few case reports describe CXL in patients with Down syndrome. Two case reports show the results of CXL performed under general anaesthesia and in both eyes simultaneously: one by Koppen et al. [21] and one by Faschinger et al. [22]. Unfortunately, these treatments resulted in severe corneal complications including corneal melting, corneal ulcer, and complicated healing. A 4-year-old patient with Down syndrome was successfully treated by CXL under general anaesthesia unilaterally; the keratoconus remained stable for 3 years [23]. A specific CXL project for patients with Down syndrome, called ‘Light for sight 21’, was founded in 2011 by Dr. Hafezi and offers a platform for research on the effects and efficacy of CXL in this patient group [24].

The likelihood for a successful CXL treatment in this specific patient group is dependent on the observed behaviour of the patient. To our knowledge, there’s no general staging of Down syndrome available. However, a valid method to estimate patient cooperation and the likelihood for a successful CXL procedure under local anaesthesia in this patient group would be valuable. Therefore, we proposed a semi-structured decision tool to help the practitioner selecting patients with Down syndrome for a CXL treatment. Some of the items, for instance ‘abrupt movements’, are considered to be a greater contraindication for CXL under local anaesthesia. Therefore, we assessed per individual patient whether CXL under local anaesthesia could be possible. Our decision tool aids in this decision. Alternatively, patients with a low score during the pre-CXL assessment could be offered the treatment under general anaesthesia.

In this case series, a prospectively selected group of patients with Down syndrome was shown. The authors are aware that only a selection of the patients visiting our outpatient clinic were shown; one other patient with Down syndrome was treated under general anaesthesia due to non-cooperation during the pre-CXL assessment and another patient with mental disability received CXL under general anaesthesia. The number of dismissed CXL treatments based on a low score on the decision tool assessment in our outpatient clinic is unknown. Although the prevalence of keratoconus in patients with Down syndrome is much higher than in the general keratoconus population, the percentage of CXL treatments in our keratoconus centre is much lower in this group.

Performing CXL to stop the progression of keratoconus and prevent a future corneal transplantation is valuable to this patient group to maintain vision and ability to function. However, an epithelial abrasion is currently still regarded essential for adequate uptake of riboflavin, since transepithelial CXL has failed to convincingly stabilize disease progression [25]. The main drawbacks with the epithelium-off technique are abrasion-related complications such as delayed wound healing and infectious keratitis. This is especially the case in mentally disabled patients who have an increased likelihood of rubbing their eyes [26].

In conclusion, this case series shows promising results of CXL under local anaesthesia in patients with keratoconus and Down syndrome. CXL should be considered in an early stage of keratoconus to avoid safety problems and premature termination of the treatment. A standardized clinical decision tool can be used for a proper patient selection to perform CXL under local anaesthesia.
